# Controlling ventricular preload using an MRI-compatible lower body negative pressure chamber: measuring changes in volumes, mechanical and hemodynamic function

**DOI:** 10.1186/1532-429X-11-S1-O77

**Published:** 2009-01-28

**Authors:** Richard Thompson, Ben Esch, Jessica Scott, June Cheng Baron, Kelvin Chow, Ian Paterson, Mark Haykowsky

**Affiliations:** 1 University of Alberta, Edmonton, AB Canada; 2 University of British Columbia, Vancouver, BC Canada

**Keywords:** Pressure Chamber, Lower Body Negative Pressure, Central Blood Volume, Gold Standard Measure, Ventricular Preload

## Introduction

Cardiac output is dependent, in part, on the ability of LV to accept preload at low filling pressure. Systematic modulation of preload is thus an important capability for the study of the preload dependence of any given aspect of cardiac performance in health and disease. Previously, LV (un)loading has been studied by control of lower body pressure, used to modulated central blood volume, primarily in conjunction with echocardiographic or invasive measures of LV volumes and systolic function[1–3]. MRI offers the gold standard measures of LV volumes and a growing number of functional parameters based on tissue and blood dynamics, but has not previously been used in conjunction with lower body pressure control. Using a low-cost custom-made MRI-compatible lower body pressure chamber we illustrate controllable preload modulation of LV volumes and mechanical and hemodynamic functional parameters (several of which have not previously been measured with variable preload).

## Methods

### MRI-Compatible Pressure Chamber

**Figure 1:**
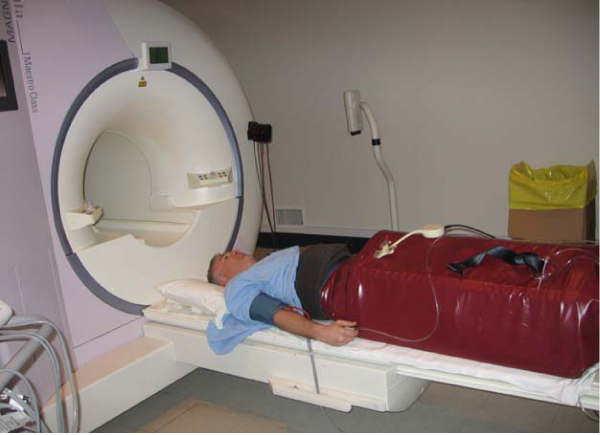
**Volunteer in the lower body pressure chamber**.

A sealed and flexible pressure chamber was constructed from a waterbed mattress using a thin flexible veneer wood frame and a kayak water skirt for subject entry (Figure 1). A compact 20 HP vacuum was used to pressurize the system with a supine subject in place (tested for pressures of -50 to 50 mmHg). 10 healthy male subjects (31 ± 9 yrs) were studied at atmospheric and -30 mmHg box pressures (serially); the later is comparable the unloading provided by standing. A 5 element cardiac receiver was used in all subjects, who were studied with conventional cines, phase contrast (LAX and SAX at base) and tissue tagging (5 SAX, 3 LAX slices) to measure a wide range of systolic and diastolic parameters; EDV, ESV, SV, EF, E and A filling waves (peak filling velocity (cm/s) and filling rate (mL/s)), intraventricular (IVPG) and atrial (IAPG) pressure gradients (calculated from LAX phase contrast data), peak systolic (S') and diastolic (E') annular velocities (cm/s), peak torsion (deg) and rate of untwisting (deg/sec), peak diastolic radial velocity (ventricular average – cm/s), and peak diastolic circumferential strain rate (ventricular average, s^-1^). All studies were breath held with ECG gating (Siemens Sonata, 1.5 T).

## Results

All subjects were comfortable throughout volume unloading experiments (~20 minutes). Tables 1, 2 and 3 summarize the changes in physiologic parameters with volume unloading. All values are reported as mean (SD). A paired t-test was used to determine if changes in parameters are significant (* p < 0.05, ** p < 0.001).

**Table 1:** Heart rate and volumes and function

	HR*	EDV(mL)**	ESV(mL)	SV(mL)**	EF(%)*
0	60.6(10.7)	177.6(28.6)	68.7(16.3)	109.0(14.8)	61.6(3.7)
-30 mmHg	63.5(9.7)	153.1(25.6)	65.8(17.1)	87.2(10.6)	57.5(4.4)

**Table 2:** Hemodynamics

	E(cm/s)**	A(cm/s)*	E_Vol_(mL/s)**	A_Vol_(mL/s)*	IVPG_peak_(mmHg)**	IAPG_peak_ (mmHg)**
0	61.6(7.7)	34.0(9.4)	567(109)	258(50)	2.9(1.2)	1.9(0.4)
-30 mmHg	46.5(10.4)	25.9(11.6)	371(57)	208(47)	1.8(0.7)	1.3(0.4)

**Table 3:** Tissue mechanics

	E' (cm/s)**	S'(cm/s)	Peak Torsion(deg)*	Peak Untwisting Rate (deg/sec)	Radial Velocity (cm/s)**	Circumferential Strain rate (s^-1^)**
0	14.7(3.1)	8.3(3.0)	11.1(2.1)	157(28)	4.4(0.9)	1.60(0.24)
-30 mmHg	9.7(2.0)	8.8(2.0)	13.4(2.8)	162(32)	3.5(0.7)	1.37(0.21)

## Conclusion

We have shown that a simple MRI-compatible lower body pressure chamber can significantly unload the LV and that a comprehensive systolic and diastolic function study is feasible during this unloading (45 minute study duration for both atmospheric and -30 mmHg unloading). Our changes in EDV and SV and standard measures of early filling (E, A and E') are comparable to previous echocardiography unloading studies[1–3] and we report significantly larger decreases in pressure gradients than previous studies[1]. To our knowledge, this is the first report of the unloading dependence of torsion (which is shown to increase with unloading), untwisting rate and radial and circumferential parameters. The superior quantitative functional imaging capabilities of MRI in combination with variable loading conditions enabled by the lower body pressure control will allow detailed physiological studies in controls and any patient group that can be studied using MRI.
